# Improvements in Surgery

**Published:** 1854-12

**Authors:** 


Improvements in Surgery. From an Introductory Address at St. Thomas' Hospital, by Samuel Solly, Esq.—Let us glance rapidly at some of our improvements. Take the motive organs of the body—bones, joints, muscles:—Caries, or ulceration of bone, whether involving the bones of the shoulder-joint, the elbow, wrist, hip, knee, or ankle-joints, used formerly to be an almost certain cause of mutilation. It was supposed that this gnawing disease, which crumbles away the bone and wastes the body, pouring forth from the crimson current of the blood, the elements of nutrition, could only be removed by the amputating knife. In the present day, we limit our operation to the removal of the disease, and preserve the sound portion of the limb. Nature proves herself a most kind and able assistant in these conservative operations. Take, for instance, the shoulder-joint. A patient comes to you with an arm, useless, painful, and debilitated from disease. He tells you—and I am now referring to no hypothetical case—that he was told in the country that he must have his limb removed, or die from the disease. He had heard of St. Thomas's Hospital in London, and he walks up, above 200 miles. He is admitted, and he has all the advantages this institution can confer. The shoulder-joint is cut into, the diseased bone is removed, the soft parts soon heal, and nature actually models for him by her absorbent vessels, a new joint, and the man returns home with an useful limb.

This description, true to the letter as regards this hospital, is, I am sure, equally true in its main features in regard to every hospital in the United Kingdom. The operation of excision of the shoulder-joint was first performed in 1769 by a provincial surgeon, Mr. White, of Manchester.

Then, again, take the elbow-joint; its structure is more complicated, but still we do not hesitate to remove the diseased parts, and in process of time a new and useful joint is formed.

This operation was first designed by Moreau, a Parisian surgeon; but we are immensely indebted to Mr. Syme, of Edinburgh, for having perfected this proceeding, and, by the frequent repetition of its performance, showing us how safe an operation it may prove in the hands of a good and skilful medical surgeon.

Mr. Anthony White, of the Westminster Hospital, in 1818, succeeded in curing disease of the hip-joint by excision of the head of the thigh bone. The second successful case was in the practice of my friend, Prof. Ferguson, of King's College; since then it has been performed sufficiently often to establish it as a recognized operation in suitable cases. I performed it a few years ago, on a patient in this hospital, with every prospect of success. The case went on well in every respect for several days, when erysipelas, that dread foe of operating surgeons, attacked my patient with fearful intensity, and he died in a few days.

From the hip-joint we may descend to the knee. This beautiful and complicated joint is, I think, more frequently the subject of that amount of severe disease requiring amputation of the limb, than any other joint in the body; but still how seldom are we obliged to amputate this joint to save life! It is indeed true that many a limb is now saved by medical surgery which formerly was sacrificed, and I must give my esteemed friend, the cod-liver oil, considerable credit for his assistance in the good cause. More than fifteen years ago, I first prescribed this medicine for strumous disease of joints, and I soon found its value. I have no hesitation in saying that we are able to carry out our conservative operations with this ally which would be quite impracticable without it.

With regard to *excision* of the knee-joint, I can give you no personal experience, but the cases which have been published encourage me to believe that it may be performed with success. And there can be no doubt that the living limb, even if shortened and the knee-joint obliterated, is more useful and more agreeable to the patient than any artificial limb, however beautifully that limb is made. I must bear my testimony to the perfection to which these mechanical contrivances are brought. This operation was first performed by Mr. Fitkin, of Northwich, and was in his hands successful.

In passing from the consideration of excision of the knee-joint to the conservative surgery of the rest of the leg, I find it impossible to tell you all that has been done. I must only attempt a rapid sketch. When I first came to this hospital in 1822, almost every amputation for disease of the leg and foot was performed a little below the knee. The value of a long lever in the use of an artificial limb was not understood, but now we save as much as possible. Amputation at the ankle-joint was never thought of. This operation was first proposed and performed in France, by Sedillier, Velpeau, and others; but the credit is due to Mr. Syme for having shown its frequent application. The retention of the firm cushion of the heel for the surface of the stump, has been of immense practical importance; and there are many men now who have been subjected to this operation who might go into any ball-room in London without the deformity being discovered. The operation is required in extensive disease of the tarsus, and it is an improvement on the old mode of amputation; but still there is an improvement upon this. Many of you have seen this carried out successfully within these walls. I refer to the removal of the diseased bones, instead of a removal of the whole foot.—*London Lancet.*

